# Ten considerations for implementing effective and sustainable near-peer teaching in clinical anatomy education

**DOI:** 10.15694/mep.2017.000087

**Published:** 2017-05-26

**Authors:** Scott Border, William Parton, Matthew Myers, Ahmed Elmansouri, Charlotte Harrison, Jonny Stephens, Eleanor Seaby, Samuel Hall

**Affiliations:** 1 University of Southampton

**Keywords:** Anatomy, Anatomy Education, Peer Teaching, Near-Peer Teaching, Medical Education

## Abstract

This article was migrated. The article was marked as recommended.

Near-peer teaching (NPT) is becoming increasing popular in medical education. The rationale and benefits of introducing such programs have been well documented and are usually described in terms of their advantages to the teacher, students and faculty. As a team that have successfully introduced two NPT anatomy programs in the last six years at the University of Southampton, we have taken a largely evidenced based approach in offering 10 considerations to ensure the implementation of a sustainable and effective NPT program in anatomical sciences. We have highlighted important aspects of NPT that will help maximise the benefit of such programs and emphasised particular areas where careful thought is necessary. We conclude that to safeguard sustainability and consistency of any given NPT program, faculty and student partnership is required, as is the maintenance of quality control and evaluative techniques.

## Introduction

There are a number of rationales for applying the practice of near-peer teaching (NPT) in medical education and its benefits have been well documented. ( Hendleman et al., 1986; Knobe et al., 2010; Nestal and Kidd, 2003; Tolsgaard et al., 2007; Colaco et al., 2006; Youdas et al., 2008; Rashid et al., 2011; Rodrigues et al., 2009; Evans and Cuffe, 2009; Bulte et al., 2007; Nnodim, 1997; Hall et al., 2012; Ten Cate et al., 2012). However, there are numerous definitions of what constitutes NPT and this can often be confusing. The key element is that the teachers are several years further along the same career pathway as the students. The varying definitions of a near-peer teacher include; “two-five years more senior”, “a student more advanced on the same curriculum” or “a student at the same level of the medical education spectrum”. Whilst variation exists regarding the scholarly relationship between teacher and learner ( Whitman and Fife, 1988), the literature widely accepts that a unique bond exists between teacher and students based on this proximity in their stage of training, and that this dynamic plays an important part in its success (Moust and Schmidt, 1994; Ten Cate et al., 2007).

In 2007, Ollie Ten Cate and Steven Durning presented twelve distinct reasons to practice NPT, and suggested that its inclusion is now becoming a deliberate element in the medical education curriculum. Their article was a comprehensive review detailing the rationales and benefits of promoting such a scheme. Prior to that Ashar Wadoodi and Joy Crosby (2002) described twelve tips informed from their own experience at Dundee. Since 2011, the anatomy centre at the University of Southampton chose to deploy NPTs to extend, broaden and diversify its range of teaching provision and to bestow all the advantages described in the 2002 and 2007 articles. Since NPT is now applied quite commonly throughout medical schools, it seemed timely to provide some guidance for obtaining the most effective NPT outcomes in clinical anatomy education and for medical education more widely.

## Consideration 1: The Curriculum – Inside or out?

*Choosing whether or not you want your NPT program to be officially part of the curriculum is an important decision. Your choice will rest entirely on the purpose of the sessions and the nature of your existing medical curriculum.*

When considering the implementation of your program as either inside or outside the curriculum, it will be worth identifying how much resistance to change will be faced by the faculty. It is possible that some academics would feel threatened by the emergence of new teachers in particular disciplines ( Whitman and Fife, 1988). Nevertheless, current student perspectives are that there should be more anatomy NPT sessions in the curriculum or it should at least stay the same ( Campolo, 2013). In modern curriculums, various guises of peer teaching are embedded in the timetable but with the emphasis being on developing the doctor as a teacher and shaping personal professional development ( Tomorrow’s Doctors, 2009). If the aim is to supplement the learner’s knowledge, it is far easier to adopt an extracurricular approach to establish the program first. A non-compulsory course driven by a student centred approach is considered an invaluable supplement to conventional teaching methods ( Lockspeiser et al., 2008; Duran et al., 2012). However, if the aim is to facilitate the development of reflective practice in whole cohorts of students as a way of meeting GMC objectives then incorporating it into the curricula would be the preferred option (McKenna and French, 2001; Campolo, 2013).

Whatever the rationale, the additional benefit of curricula driven NPT is that of forthcoming resources; most notably the support and recognition of the medical school ( Wadoodi and Crosby, 2002). An extracurricular approach provides more flexibility with delivery but at the expense of status and perceived value to the cohort. At Southampton, we decided to move our neuroanatomy NPT program from outside to inside the curriculum after two years and witnessed significant levels of knowledge gain in pre and post testing as well as a rise in the overall module evaluation scores. There is little doubt that including a well organised NPT program inside the curriculum in subject specific areas enhances the student learning experience ( Brueckner and MacPherson, 2004; Krych et al., 2005; Stephens et al., 2016a).

## Consideration 2: Choosing appropriate medical topics – can you near-peer teach anything?

*You need to think why your particular subject area could benefit from NPT. There should be recognisable benefits to the faculty, the teachers and the learners. If there is, there is a good chance NPT is the right educational development to meet your needs.*

Whilst the application of NPT has been successful in a range of scenarios, it appears to be particularly prevalent amongst the health professions and life sciences ( Youdas et al., 2008; Rodrigues et al., 2009; McKenna et al., 2011; Naeger et al., 2013; Tayler et al., 2015). It does, of course, have a long and established history in the anatomical sciences ( Hendleman et al., 1986; Krych et al., 2005; Bentley and Bulte et al., 2007; Evans and Cuffe, 2009; Hill, 2009; Duran et al., 2012; Sugand et al., 2016; Ocel et al., 2016).

The shift in focus towards professional led medical curricula has reduced the amount of time allocated to teach the basic sciences in the UK. Clinical anatomy in particular has experienced significant reductions in dedicated teaching hours, raising concern over the adequacy of anatomical knowledge necessary for clinical practice ( Heylings, 2002; Gogalniceanu et al., 2009). The volume and pace of taught material in medical training increases the likelihood that students will fail to meet all the learning outcomes expected of them. As a result, they may have many unmet educational needs in a range of disciplines that could benefit from NPT.

In many examples, in the USA, the aim of implementing NPT has been to alleviate teaching pressures on the faculty and to compensate for growing cohort sizes, especially to meet the demands of small group teaching ( Cate and Durning, 2007). The existing evidence suggests that providing additional teachers in the form of senior medical students does not compromise student learning ( Tolsgaard et al., 2007; Burke et al., 2007). The wider impact however may not have been considered; market-led Universities in British higher education are more frequently responding to consumer calls and focusing on what students want - currently, a higher staff/student ratio and more personalised content delivery ( Molesworth, 2009). higher education institutions should therefore carefully consider their motives if they wish to avoid being accused of exploiting NPTs for financial purposes.

Our view is focused upon consideration of subject specific NPT, and the recommendation of using it in a setting where education can be enhanced by the skill of the individuals involved, rather than to increase the frequency of popular and expensive teaching methods. This tailored and targeted approach of NPT is supported by the work of Rodrigues et al., 2009. We are also in agreement with Wadoodi and Crosby 2002 in that NPT is particularly good for integrating learning if the peer teachers are in clinical years of study and the learners are not. In our experience, neuroanatomy is a topic that works very well; it is complex and conceptual, and students frequently struggle with it ( Fantaneanu et al., 2014). Therefore, it lends itself to being understood more deeply when explained in different ways because of the steeper learning curves involved ( Hall et al., 2015). This gives more scope to maximise the benefits of NPT and harness the potential of social and cognitive congruence which inevitably lead to breakthroughs of understanding ( Stephens et al., 2016a). In a recent survey of students at Southampton, pelvic anatomy was rated as the most difficult anatomical area to learn besides the brain due to its complex three dimensional arrangements ( Hall et al., 2015). This suggests it would be a good choice for NPT participation. However, at a recent national workshop comprising members of the British Association of Clinical Anatomists and The Anatomical Society of Great Britain and Ireland ( Border et al., 2017) (N=78) the majority of delegates (66%) felt they would most like to implement NPT in musculoskeletal anatomy classes ( Stephens et al., 2016b). Interestingly, this decision was based on content that NPTs would find easiest to teach rather than the topic that would be best suited to the learner’s needs. Ultimately, NPT can be used ubiquitously to enhance the student experience but gains in knowledge compared with faculty teaching are probably negligible with more straight forward subject matter.

## Consideration 3: Teaching strategy – make the purpose of the session clear to the learners

*The approach you take to the teaching will depend on many things; however, it helps if there is a definitive purpose to the sessions creating consistency between the material taught and the assessment type.*

Over time, there has been a pedagogic shift from the long established teacher centred approach to a student centred one where the emphasis is more upon the students and what they learn ( Spencer and Jordan, 1999). The structure of UK medical curricula is conducive to a surface learning approach because students have a heavy workload, where there is a vast amount of course material to master and seldom opportunity to study subjects in depth or revisit them ( Spencer and Jordan, 1999).

We believe that a NPT program can help nurture deep learning because in the NPT environment learners are enthused by material they need to know and how it is integrated in clinical practice ( Smith and Mathias, 2010). The emphasis is still placed on teacher knowledge but it becomes more important for NPT’s to appreciate how they have learned and how others build knowledge just as they have done. In order to evaluate the effectiveness of tailored NPT programs, to some degree coordinators are forced to foster an outcome/ performance based approach as a means to objectively measure their utility. However, the approach to teaching tends to be student centred, naturally because the teachers themselves are students.

## Consideration 4: Selecting student teachers – select them rather than them selecting you

*The student teachers are the lifeblood of any NPT program. Harnessing their passion and enthusiasm for the subject is what gives NPT its inimitable advantages.*

The GMC has a number of overarching outcomes for graduates in accordance with its Tomorrow’s Doctors mandate. Included as part of the umbrella term ‘the doctor as a professional’ are standards relating to reflection, learning and teaching. As such graduates of medical programs should be able to function effectively as a mentor and teacher ( Tomorrow’s Doctors, 2009). Since most students are familiar with the importance of this publication, acquiring motivated individuals appreciative of the teaching experience requires minimal effort. In many cases, NPT program coordinators take the common option of generating an e-mail advert with a subsequent criterion led selection process ( Campolo et al., 2013). According to Wadoodi and Crosby (2002), the equal opportunity method is preferable otherwise the faculty might be viewed as exploiting student’s skills.

The alternative option, and the one we chose at Southampton, is to identify students with the most potential, approaching only the ones who have been observed to demonstrate high attendance, motivation and passion for the subject in formal sessions. The proposition is that those who have a love of a subject will not only communicate information enthusiastically and more effectively, but are motivated by the opportunity rather than purely for reasons aligned to fulfilling criteria for their own portfolios.

The more motivated students are likely to contribute more regularly and be capable of investing in a scholarly approach to teaching, which can offer more in the way of reward ( Hall et al., 2013). Furthermore, it dispels any apprehension on the part of the learner about trusting the taught content, which has been an issue with some programs ( Bulte et al., 2007). A significant drawback to this approach is that it does not naturally select those who demonstrate specific teaching qualities since it is more focused on subject knowledge. This is much less of a problem if there is true student faculty collaboration; the role of the faculty is there in part to foster good teaching practice and encourage reflection. If the students receive scholarly rewards they are likely to continue to invest their time over more than a single session. As your student teachers gain more experience their teaching performances improve and the program is more likely to become sustainable.

## Consideration 5: Create a partnership between yourself and the students – Offer trust and take risks, the rewards will pay off

*Success is dependent on establishing a trusting relationship where you can model accountability and ownership of the program.*

Traditionally the term which describes staff and students working together is called supervision, presumably because the faculty member holds a position of authority and is the one who guides and advises the student. Mentorship is the relationship between a more experienced professional and a protégé - the mentor is a trusted advisor, and this type of relationship offers many advantages within NPT which are summarised below.

• It encourages loyalty
• It increases morale
• It stimulates creativity
• It promotes a sense of trust

At Southampton we strongly promote a collaborative approach which adopts a mentorship style of working within a partnership. We believe the success of our program is due to a work ethic where all ideas have equal value, no matter the experience of the contributor. This is very much in line with the Higher Education Academy’s toolkit for working with students as partners in learning and teaching ( Healey, 2014). True collaboration creates the appropriate dynamics to allow for the freedom of thought and expression. This way of working implies the crossing of hierarchical boundaries which not all academics are comfortable with. Understandably, some staff have concerns that act as a barrier to partnership, such as quality control and ownership of the project ( Bovill et al., 2014). Despite this, a balanced partnership leads to increased levels of engagement and a deeper meta-cognitive understanding of learning and teaching processes ( Cook-Sather et al., 2014), in this type of setting, we believe an NPT program is much more likely to flourish and be sustainable for the future.

## Consideration 6: Think about the timing of delivery

*Understanding how students perceive and value teaching is essential to the timing of delivery. Use your student teachers insight to pitch the sessions to your audience at a time when they will need it most.*

NPT initiatives can be implemented as front line teaching where students are learning subject information for the very first time within the curriculum. Alternatively, NPT can be deployed as a supplementary aid, delivered after the faculty have provided the module/course content. The former is usually hierarchical, sometimes contractual, and mostly conventional, rendering the relationship between staff and student’s to be much like that of student and faculty member. It is likely that contractual relationships force the NPT to behave more like a staff member than a student which affects the teaching dynamics and social congruence.

The supplementary approach cultivates a less pressured delivery while also promoting a more natural learning environment. Presumably this is because there is less expectation. When students already have a basic understanding of a topic before the session, but find certain principles difficult to master, NPT is ideally placed to offer alternative explanations and approaches to learning through the student teacher’s own style. Moreover, the sessions can be centred on known problem areas (known to the NPT because they have been there themselves). It is our belief that near-peers should have the freedom to relate to students in a way that is consistent with their natural approaches and personality, but when they are formally involved in faculty teaching delivery, the full benefits of NPT might be more difficult to achieve. When UK anatomists were asked their preference at a national workshop ( Border et al., 2017), (N=78) only 4% of attendees were in favour of using NPT exclusively for frontline teaching. The majority preferred the option of having either revision (supplementary) sessions (32%) or a mixture of both (62%). Delegates felt that frontline delivery in isolation was a high risk option unless students were experienced and well trained ( Stephens et al., 2016b). More recently, findings from our own research at Southampton have demonstrated that student experience ratings are significantly higher when NPT is frontline ( Border et al., 2017). Knowledge gain is also greater but this is to be expected since baseline knowledge at the start of the module is low. If you are confident in your teacher’s ability, moving some aspects of NPT delivery to the beginning of the module is very worthy of consideration, particularly where subject matter is difficult and some repetition is desirable. Most importantly the opinions of your NPTs are of paramount importance; they will have a better insight than you as to what students need and when they need it.

## Consideration 7: Select the most effective educational distance between your student teachers and learners; it is important to maintain the right level of congruence.

Social and cognitive congruence are often considered to be the main strengths of NPT ( Hall et al., 2014). This is formed by the proximity in stages of training between student and teacher (Schmidt and Moust, 1985; Lockspeiser et al., 2008). In a previous article we argued that levels of congruence between the teacher and learner are likely to change as the distance is increased along the near-peer teaching spectrum ( Stephens et al., 2016a). In particular, we highlighted student’s perceptions of criteria such as enjoyment levels, use of teaching time and delivery of content as being especially sensitive to increases in educational distance between tutor and learner. Senior medical students are preferred to junior doctors as near-peer teachers in our early year program and this has a significant impact on student’s perception of their own knowledge gain ( Hall et al., 2016). It is highly probable that the sensitivity of distance along the NPT spectrum may vary from discipline to discipline, making it difficult to define an optimum distance. If there is too much congruence, (as with peer rather than near peer teaching), the teaching becomes more collaborative in nature ( Jackson and Evans, 2012). Furthermore, there is also evidence of tension and competitiveness when students are involved in teaching their own cohort ( Parton et al., 2017). It is clear that the dynamics are better when some distance exists. Our research suggests that congruence begins to fade when the tutor is beyond a point of two years further on in their training. As the student teachers progress closer to becoming a doctor, their focus may change and their teaching is less well aligned to curriculum outcomes. Lack of time to prepare due to added pressures in the workplace may also be a factor to consider. Junior doctors are often working long hours with varied shift patterns leaving them tired and fatigued which may negatively impact upon teaching performance.

## Consideration 8: Sustainability ensures the greatest reward

*There is only one thing more difficult than establishing your NPT program; sustaining it. As the students develop, mature and ultimately move on, use them to inspire and train the next generation of near-peer teachers.*

Near-peer teaching is frequently referred to as having a number of benefits to the student teachers (Duran et al., 2012; Dandavino et al., 2007; Lockspeiser et al., 2008). Not only do they learn the subject material in greater depth and get a positive feeling of helping other students, they also acquire a myriad of transferrable skills which collectively constitute professional development. With repeated delivery of the program comes experience, and with experience comes cognitive growth - the art of restructuring new knowledge with what is already known ( Biggs, 2011). If you only let your NPTs teach once, they won’t get the full benefit from it and nor will they deliver their best teaching to the students.

We believe that the true benefit of sustainability is an opportunity for NPTs to develop an element of scholarship into their teaching. Ultimately, this will enable them to generate original contributions of knowledge to the field and inform future practice. Students that do so are rewarded with the opportunity to present their work and contribute towards manuscript preparation. During our national workshop with a range of UK anatomists ( Border et al., 2017), 36% believed this to be the most appropriate method of reward for NPTs. 38% would prefer to reward students through an official accredited system, such as certification ( Stephens et al., 2016b). Harnessing the power of intrinsic motivators in the first instance is what will provide you with the right NPT candidates, but offering longer term academic rewards will likely deepen and sustain student-staff partnerships ( Bovill et al., 2014).

When thinking about how to improve each session, the NPT can do so through reflective practice, while the mentor can encourage sensible risk taking with aspects of teaching such as structure and delivery ( Hatton and Smith, 1995). Peer directed learning can enrich learning outcomes ( Collier, 1983) and over time, experienced NPTs will become aware of what these are and try to promote them through refinement of their teaching habits. The principles of good teaching practice can then be grounded in the next generation of NPTs, initially through observation, and then encouraged through contributions to session design and structure. The newly recruited teachers will not only have had an opportunity to build their subject knowledge but will be well versed in a self-monitoring approach towards developing their teaching practice ( Kolb, 2014). In our view this will offer a level of quality assurance, consistency and sustainability to the program that cannot be achieved by faculty support alone.

## Consideration 9: Ensure quality control and reputation

*Much of the peer teaching in medical schools takes place in the hidden curriculum - an underground movement of students who want to support each other and reinforce core knowledge. The faculty is in a good position to channel this enthusiasm and offer these students the quality control they need.*

In 2007, Ten Cate and Durning provided an audit of the literature where they listed manifestations of peer teaching along with its rationale for use and educational distance between teacher and learner. They included a formality rating which can be interpreted as institutional recognition and approval of the scheme. As expected, from the 28 citations listed nearly all offered a level of formality. It is speculated that many more examples of NPT exist within the hidden curriculum but go unreported ( Havnes, 2008). Evidence of their organisation can be found through social media and through student societies at institutional level.

One major advantage of faculty involvement in the program is authorised use of teaching resources and materials. Liaison with the faculty allows for an opportunity to confirm understanding with student teachers. Despite this, some faculty staff are understandably unhappy with students using their teaching resources, particularly where the presentation slides contain intellectual property.

## Consideration 10: Adopting a scholarly approach is good practice

*Embedding scholarship into teaching practice allows near-peer teachers to appreciate research led teaching and to potentially make original contributions of their own. Investing in more than just the participation of teaching strengthens the commitment of the students, thus providing an incentive beyond their original interest in the taught material.*

The importance of research alongside teaching is a central value to higher education. As scholars, your NPT’s are more than just student teachers; they can be encouraged to be researchers, and as such become naturally inquisitive about the processes that drive learning in their sessions ( Healey and Jenkins, 2006). In our experience, an established and sustainable NPT program is much more conducive to meeting the aims and objectives of the research questions that you might have about your program. Teaching scenarios can be manipulated to test hypotheses or compare teachers or student groups. Longitudinal studies can be introduced in the safe knowledge that several years’ worth of data will be forthcoming. Furthermore, the students themselves are given the opportunity to collect, analyse, interpret and disseminate the findings with support from the faculty ( Stephens et al., 2016a).

The perceptions of student learners are useful in evaluating the program but it is also important that being in attendance at the sessions is effective in the transfer of knowledge ( Wong et al., 2007). In a comprehensive retrospective study Ten Cate et al (2012) concluded that near peer tutoring has similar benefits to faculty teaching in terms of academic achievement. This study, like a few others in the field, compared end of module examination scores, although a common alternative approach is to measure knowledge gain via pre and post testing (Topping, 1996; Wong et al., 2007; Ten Cate et al., 2012; Rees et al., 2016). Surprisingly, only a small number of published studies on NPT in anatomy have investigated the impact of NPT on student learning outcomes. Furthermore, it is important when considering the effectiveness of NPT as an intervention, that its success is not attributed to confounding factors. The most notable ones are class size, age of the teacher and experience of the teacher.

## Conclusion

Near peer teaching has some well-known advantages making it increasingly important to investigate how to capitalize on its potential impact within higher education. The current article makes a case for why a sustainable program offers the best opportunity to maximise outcomes for all parties. However, the considerations discussed above are certainly not exhaustive. For example, we have not discussed what sort of training to offer NPTs or the type of learning environment (lecture theatres, tutorials or practical sessions) to choose. Furthermore, this article has opted not to discuss some of the tensions that may result when adopting certain aspects of this model, such as ownership of the program and the huge organisational and administrative burden placed on academic staff leading the initiative. To this end, it is recognised that in some areas of teaching NPT led instruction might not be cost effective or realistic.

Therefore, it is important that when NPT is implemented it is done so for the right reasons and in the most effective way. The investment of time and effort required to making it successful is considerable, so making choices that support sustainability are crucial. In our experience of running a successful NPT program in neuroanatomy at Southampton for six years, the take home message is, that working with students as partners breaks down existing barriers of hierarchy and enables the best possible chance of success.

## Notes On Contributors

Scott Border B.Sc., (Hons) Ph.D., F.H.E.A., is a Principal Teaching Fellow in Anatomy at the Centre for Learning Anatomical Sciences and Deputy Head of the Academic Unit, Medical Education. He is subject lead for Neuroanatomy and his research interests are in medical education, neuroanatomy, peer-teaching and learning technologies.

William Parton is a fourth year medical student at The University of Southampton. He conducted his M.Med.Sc. research on near-peer teaching in neuroanatomy and is an experienced near-peer teacher who coordinates the training of future student teachers.

Matthew Myers is a fourth year medical student at the University of Southampton. He conducted his M.Med.Sc. research in clinical neuroscience. He is an experienced near-peer teacher who coordinates the training of future student teachers and contributes to educational research.

Ahmed Elmansouri is a fourth year medical student at the University of Southampton. He is an experienced near-peer teacher who coordinates the training for future student teachers and contributes to educational research.

Charlotte Harrison is a fourth year medical student at the University of Southampton. She has conducted her M.Med.Sc. research in clinical neuroscience. She is an experienced near-peer teacher who coordinates the training of future student teachers and contributes to educational research.

Jonny Stephens M.B.B.S., M.Med.Sc., is a foundation year 2 doctor at St Mary’s Hospital London and Visiting Fellow at the Centre for Learning Anatomical Sciences, University of Southampton. He is founding member of the near-peer teaching program and conducts educational research around near-peer teaching.

Eleanor Seaby M.B.B.S., M.Med.Sc., is a foundation year 1 doctor and Visiting Fellow of the Department of Human Genetics and Genomic Medicine at The University of Southampton. She is an experienced teacher and former coordinator of the near-peer teaching program at Southampton where she also contributed to educational research.

Samuel Hall BM., M.Med.Sc., is a year 2 neurosurgical trainee at University Hospital Southampton and a Visiting Fellow at the Centre for Learning Anatomical Sciences, University of Southampton. He is a founding member of the near-peer teaching program and has led on a range of educational research initiatives in neuroanatomy education and near-peer teaching.
