# Supplementary material for: Ten considerations for implementing effective and sustainable near-peer teaching in clinical anatomy education
Source: MedEdPublish (2016). 2017 May 26;6:87. [Version 1] doi: 10.15694/mep.2017.000087 (PMC10885257; doi:10.15694/mep.2017.000087)
Supplement: Peer review history

# Reviewer report: Reviewer response for version 1

**DOI:** 10.21956/mep.19253.r27955

**Published:** 2018-06-15

**Authors:** Anthony David.M

**Affiliations:** 1 Soho

This review has been migrated. The reviewer awarded 4 stars out of 5 A very informative paper which throws light on several considerations for Near Peer Teaching. Our experience has been in Peer Teaching in the form of Student led seminars. Many students excelled in communicating well with their peers. The peers also gave a good feedback on this. All the ten considerations enumerated by the authors are well put and are pragmatic. Ensuring quality control and reputation of these programs is difficult but essential to further such programs. Thank you authors for this paper.

Reviewer Expertise:

NA

No decision status is available

# Reviewer report: Reviewer response for version 1

**DOI:** 10.21956/mep.19253.r27954

**Published:** 2017-06-13

**Authors:** Madalena Folque Patricio

**Affiliations:** 1 School of Medicine, University of Lisbon

This review has been migrated. The reviewer awarded 4 stars out of 5 A very god paper on 10 approaches to be considered when implementing near-peer teaching (NPT). Reading this article was particularly helpful because NPT is in place at my school (the Faculty of Medicine of the University of Lisbon) in Clinical Anatomy as well as in other teaching areas, This possibility is offered as an elective during a full semester with senior students collaborating with the teaching team. Great advantages are recognized by teachers and students and the 10 considerations presented by the authors will be a wonderful guide to revisit and improve what we are doing at home. This is why I highly recommend this paper not only for those who want to implement such a teaching approach but also to those who are already doing something similar but looking for improvement. I can’t be more in agreement with the authors´ conclusion when they say that ‘working with students as partners breaks down existing barriers of hierarchy and enables the best possible chance of success’.Before concluding I would like to suggest something to the authors of this excellent paper namely to also report on the less positive aspects, difficulties and barriers (if any) and how they were overcome.

Reviewer Expertise:

NA

No decision status is available

# Reviewer report: Reviewer response for version 1

**DOI:** 10.21956/mep.19253.r27956

**Published:** 2017-06-01

**Authors:** Susmita Reddy Karri

**Affiliations:** 1 Topiwala national medical college and BYL nair charitable hospital, mumbai. india

This review has been migrated. The reviewer awarded 4 stars out of 5 A very good paper on the successful implementation of NPT. However, some of the points are not just 'exclusive' for NPT , they can be generalised. But overall, a very good and balanced paper which focuses on both the positives and negatives.

Reviewer Expertise:

NA

No decision status is available

# Reviewer report: Reviewer response for version 1

**DOI:** 10.21956/mep.19253.r27959

**Published:** 2017-05-30

**Authors:** Julie Browne

**Affiliations:** 1 Cardiff University School of Medicine

This review has been migrated. The reviewer awarded 3 stars out of 5 I enjoyed this paper and think it will be of value to readers, especially those who are in the early stages of planning to introduce it. It is easy to read and the ‘ten considerations’ structure works really well. It is always good to hear from authors who have practice experience at the cutting edge of teaching and who can advise on the benefits and pitfalls. For me it raised two interesting questions: (1) Were the ten considerations in order of importance, in chronological order of ‘things to do when getting started’, or some other order? Either way, I would suggest that adopting a scholarly approach deserves a higher priority. (2) Which of these considerations are unique to peer teaching, and which are generic to all teaching? Quality control and setting learning outcomes, are, I would argue, essential and non-negotiable for all medical teaching, whereas the authors rightly point out that there are unique challenges in selecting and briefing peer teachers.

Reviewer Expertise:

NA

No decision status is available

# Reviewer report: Reviewer response for version 1

**DOI:** 10.21956/mep.19253.r27952

**Published:** 2017-05-30

**Authors:** Barbara Jennings

**Affiliations:** 1 Norwich Medical School

This review has been migrated. The reviewer awarded 4 stars out of 5 In this article by Scott Border et al., the successful application of Near-Peer Tutoring (NPT) within the medical school’s Anatomy curriculum is described, and its evidence-base is presented in their very useful introduction. This type of manuscript (Practical Tips & Guidelines) offers the benefit of real-world experience to the wider community of practice, and I also thought that the authors’ thorough use of citations about the evidence base of NPT was a real strength in this paper. The tips presented were nuanced, describing points of caution as well as positive experiences. I found the messages about the importance of creating a sustainable curriculum particularly resonant; we have all experienced the zeal and enthusiasm that accompanies the introduction of an initiative that is in vogue, but this can rapidly dissipate when multiple iterations are required. Using a strategy to pass on knowledge and training, and one that encourages scholarship and reflective practice too sounds like an approach designed for long term success.A couple of suggestions/questions for the authors: 1. It is worth checking manuscripts for both syntax problems and typos (there are a few here) before final submission – this is particularly important before submitting to journals like MedEdPublish that use post-publication-peer-review.2. I wondered about your selection strategy for NP tutors. It was unclear to me about how you identify potential candidates; how do you select based on motivation and passion objectively? One advantage of a criterion-based selection process with wide advertising is that you can select from a wide pool of folk attracted for a range of reasons. Have you checked that the demography of your NP tutor cohort is representative of the wider student population?In our opening editorial we wanted to encourage the submission of articles that described student partnerships and the development of transferable skills through the delivery of the Life Sciences Curriculum. This article was therefore very welcome; and it is particularly encouraging to see that the authors include both undergraduates and recent medical school graduates, who have contributed to the NPT design and delivery faculty-team at the University of Southampton.

Reviewer Expertise:

NA

No decision status is available

# Reviewer report: Reviewer response for version 1

**DOI:** 10.21956/mep.19253.r27953

**Published:** 2017-05-28

**Authors:** Michael SH Wan

**Affiliations:** 1 University of Notre Dame

This review has been migrated. The reviewer awarded 4 stars out of 5 A very well written review on the 10 considerations in NPT! This provide a very good foundation for any institution considering this approach to Anatomy teaching; either as part of the formal curriculum or as a supplement to student learning, Continual evaluation of the program by both the students and tutors/teachers are very useful for the continual improvement. I agree with the point that NPT tutor should preferably been selected by the faculty. This article will spark more schools to consider this form of teaching in Anatomy as well as other disciplines.

Reviewer Expertise:

NA

No decision status is available

# Reviewer report: Reviewer response for version 1

**DOI:** 10.21956/mep.19253.r27951

**Published:** 2017-05-28

**Authors:** Dujeepa D. Samarasekera

**Affiliations:** 1 National University of Singapore

This review has been migrated. The reviewer awarded 4 stars out of 5 Excellent paper providing useful information how best to incorporate NPT. Recommend this to med educators who are planning to develop and or encourage NPT in their institutions or learning environments.

Reviewer Expertise:

NA

No decision status is available

# Reviewer report: Reviewer response for version 1

**DOI:** 10.21956/mep.19253.r27958

**Published:** 2017-05-27

**Authors:** BALAJI ARUMUGAM

**Affiliations:** 1 TAGORE MEDICAL COLLEGE AND HOSPITAL

This review has been migrated. The reviewer awarded 4 stars out of 5 Congrats to the authors for publishing such a worth reading article on NPT which is a very difficult area in medical education. As the author discussed the ten key areas for considering the NPT implementation, the most common advantages and disadvantages of NPT also should have been dealt with so that the article would have been more comprehensive. But still I personally feel that this particular article has a great content divided into 10 important key areas for the implementation of NPT. Good Job - Scott Border et al...

Reviewer Expertise:

NA

No decision status is available

# Reviewer report: Reviewer response for version 1

**DOI:** 10.21956/mep.19253.r27957

**Published:** 2017-05-26

**Authors:** Trevor Gibbs

**Affiliations:** 1 AMEE

This review has been migrated. The reviewer awarded 4 stars out of 5 As the authors state, near-peer teaching (NPT) is spoken about a lot, but its practice dos tend towards the "hit and miss". A structured approach is necessary and this paper provides a very well balanced, well referenced, approach to the subject. I particularly enjoyed reading the relevant 10 issues related to NPL and liked the balanced approach which still left more room for discussion.I would think that this paper would be a valuable read to anyone who intends to introduce NPT, whilst also providing a very solid background to the topic

Reviewer Expertise:

NA

No decision status is available
